# Effects of Factor XIII Deficiency on Thromboelastography. Thromboelastography with Calcium and Streptokinase Addition is more Sensitive than Solubility Tests

**DOI:** 10.4084/MJHID.2016.037

**Published:** 2016-08-16

**Authors:** M. Martinuzzo, L. Barrera, D. Altuna, F. Tisi Baña, J. Bieti, Q. Amigo, M. D’Adamo, M.S. López, J. Oyhamburu, J.C. Otaso

**Affiliations:** 1 Grupo Bioquímico. Laboratorio Central del Hospital Italiano de Buenos Aires; 2 Instituto Universitario del Hospital Italiano; 3 Servicio de Hematología y Oncología Pediátrica. Transplante de Médula Ósea del Hospital Italiano de Buenos Aires; 4 Servicio de Pediatría y Hematología del Hospital Iturraspe de Santa Fe

## Abstract

**Background:**

Homozygous or double heterozygous factor XIII (FXIII) deficiency is characterized by soft tissue hematomas, intracranial and delayed spontaneous bleeding. Alterations of thromboelastography (TEG) parameters in these patients have been reported. The aim of the study was to show results of TEG, TEG Lysis (Lys 60) induced by subthreshold concentrations of streptokinase (SK), and to compare them to the clot solubility studies results in samples of a 1-year-old girl with homozygous or double heterozygous FXIII deficiency.

**Case:**

A year one girl with a history of bleeding from the umbilical cord. During her first year of life, several hematomas appeared in soft upper limb tissue after punctures for vaccination and a gluteal hematoma. One additional sample of a heterozygous patient and three samples of acquired FXIII deficiency were also evaluated.

**Materials and Methods:**

Clotting tests, von Willebrand factor (vWF) antigen and activity, plasma FXIII-A subunit (pFXIII-A) were measured by an immunoturbidimetric assay in a photo-optical coagulometer. Solubility tests were performed with Ca^2+^-5 M urea and thrombin-2% acetic acid. Basal and post-FXIII concentrate infusion samples were studied. TEG was performed with CaCl_2_ or CaCl_2_ + SK (3.2 U/mL) in a Thromboelastograph.

**Results:**

Prothrombin time (PT), activated partial thromboplastin time (APTT), thrombin time, fibrinogen, factor VIIIc, vWF, and platelet aggregation were normal. Antigenic pFXIII-A subunit was < 2%. TEG, evaluated at diagnosis and post FXIII concentrate infusion (pFXIII-A= 37%), presented a normal reaction time (R), 8 min, prolonged k (14 and 11min respectively), a low Maximum-Amplitude (MA) ( 39 and 52 mm respectively), and Clot Lysis (Lys60) slightly increased (23 and 30% respectively). In the sample at diagnosis, clot solubility was abnormal, 50 and 45 min with Ca-Urea and thrombin-acetic acid, respectively, but normal (>16 hours) 1-day post-FXIII infusion. Analysis of FXIII deficient and normal plasma mixtures (< 2–102% of pFXIII-A), showed that Ca-urea solubility was abnormal at pFXIII-A < 9%, thrombin-acetic acid at pFXIII-A<18%, but TEG MA and elasticity at 23% and Lys60 with SK at pFXIII-A< 40%.

**Conclusions:**

TEG parameters MA and elasticity, and Lys 60 in TEG either with Ca^2+^ or Ca^2+^ and SK are more sensitive to low levels of pFXIII than solubility tests. The increased Lys60 induced by a subthreshold concentration of SK could probably reflect the clot characteristics “in vivo” in many patients with pFXIII levels between 5–40% and could be potentially considered as screening test.

## Introduction

Factor XIII (FXIII) is the fibrin stabilizing factor that circulates in plasma as a heterodimer with two catalytic A- subunits and two carrier B- subunits, with different sites of production: bone marrow megakaryocytes and monocyte/macrophage cell lines for FXIII-A and liver for FXIII-B. Upon thrombin cleavage of Arg37-Gly38 in the A- subunit and calcium presence B- subunit dissociates from A subunit, the cysteine active site (Cys314 interacting with His373 and Asp396) in the central core is exposed and FXIII-A becomes activated. FXIII-A has a transglutaminase action that produces a gamma glutamyl epsilon lysine crosslinking between fibrin fibers, stabilizing and conferring them viscoelastic resistance to shear forces and also to increase the resistance to fibrinolysis by plasmin, due to the crosslinking of fibrin and α2 antiplasmin.^1^–^3^

Most congenital FXIII deficiencies are a result of FXIII-A-subunit deficiency, and the A subunit is absent from plasma, platelets, and monocytes. On the other hand, congenital deficiency in the FXIII-B subunit is a relatively rare cause of FXIII deficiency, and levels of the B subunit are usually reduced, and very rarely both A and B subunits are absent. Bleeding symptoms in patients with FXIII-B deficiencies are relatively milder than FXIII-A deficiencies.^4^–^6^

For a long time, it was thought that a level of 5% plasma FXIII (pFXIII) was enough for efficient hemostasis. However, there is increasing evidence that more than 50% of patients with low pFXIII levels, but higher than 5%, could have bleeding complications after surgery, trauma or dental extraction^7^,^8^ because of a heterozygous deficiency condition, affecting either A or B subunits. Moreover, the patients with either congenital heterozygous or acquired FXIII deficiency, due to consumption or autoantibody inactivation, experience bleeding.^9^–^12^

The clot solubility test is an inexpensive procedure that has been used in hemostasis laboratories, but it has the limitation of detecting only homozygous or double heterozygous FXIII deficiency.^6^,^9^,^13^ Quantitative assays of FXIII activity and antigen are now recommended by experts because they can detect heterozygous congenital as well as mild acquired deficiencies, automate instruments can be used and then they can be better standardized.^6^ Furthermore, viscoelastic and lysis characteristics of the clot in patients with FXIII deficiency could also be important to explain clinical manifestation, even in patients with heterozygous congenital or mild acquired deficiencies.

In this report, we present a case of Congenital FXIII deficiency (with pFXIII levels compatible with homozygous or double heterozygous deficiency) and the effects of FXIII deficiency on thromboelastography (TEG). The aim was to assess the sensitivity of TEG parameters, particularly lysis (Lys 60) to the addition of subthreshold concentration of streptokinase (SK) to different concentrations of FXIII and compare it with classical screening solubility tests.

## Material and Methods

### Patients

We studied a 1-year-old girl who was referred to our hospital because of excessive bleeding. She had a history of umbilical cord bleeding. During her first year of life, several spontaneous bruising and post-vaccination muscle hematoma appeared. When she started to walk a fall from her altitude generated an extensive gluteal hematoma. After a diagnosis of a severe FXIII deficiency (with pXIII-A levels compatible with homozygous or heterozygous deficiency), the patient was started on a prophylactic plasma-derived FXIII (Fibrogammin P) therapeutic at 10U/Kg of body weight every 4 weeks. A FXIII-A control baseline and a post-infusion were performed.

### Additional Patients

We included studies of one mild congenital (possibly heterozygous) and three acquired post-surgery deficient patients’ samples, to evaluate the effect of FXIII on the clot and lysis parameters on TEG and TEG with SK. Patients presented fibrinogen levels between 180–360 mg/dL, and suffered late severe bleeding complications. Two female patients (56 and 68 years old) underwent major abdominal surgery, bowel surgery, complicated with bleeding and received, red cells, platelets, plasma and one of them, cryoprecipitate until pFXIII-A measurement was ordered. The third patient (male, 62 years old) underwent a cardiac bypass surgery with a very prolonged extracorporeal circulation time. This patient received red cells, platelets, plasma and cryoprecipitate until pFXIII measurement was made. Samples were taken between day 1–3 post surgery, and one patient needed three reoperation because of bleeding until his pFXIII was measured and replaced. All patients stopped bleeding and improved after FXIII replacement therapy.

### Normal controls

Five healthy donors’ samples (3 females and 2 males) from the laboratory staff were used as normal controls. Each one was used as a control on every working day, to check the whole blood TEG with Ca^2+^ and with Ca^2+^ + SK. For plasma tests, a pool of the 5 plasmas was used in plasma mixtures experiments.

### Methods

Blood collection: Blood was drawn from antecubital vein into 0.11M Sodium Citrated tubes (BD Vacutainer® Blood Collection Tubes, Becton, Dickinson and Company, Franklin Lakes, NJ, USA ), at diagnosis, at 24 hours of the first replacement therapy and 21 days after the last replacement, 12 months from replacement therapy start.

Prothrombin time [PT Fibrinogen HS Plus, Instrumentation Laboratory, Bedford MA, USA, (IL)], activated partial thromboplastin time (APTT: HemosIL APTT SP, IL), Fibrinogen (Fibrinogen C, IL), Factor VIIIc by a one stage coagulometric assay, von Willebrand factor (vWF) antigen (vWF:Ag, HemosIL VWF Antigen, IL), vWF activity (HemosIL VWF Activity, IL) and FXIII-A subunit by immunoturbidimetry (IL) were performed in a ACL TOP 700 coagulometer (IL). For platelet aggregation study, ADP, Epinephrin, Collagen and arachidonic acid (Helena Laboratories, Beaumont, Texas USA) as agonists and a dual Chanel light transmission aggregometer (Chrono-Log, Harvest on, PA, USA) were used. Platelet adhesion and function with COL/EPI and COL/ADP cartridge were run on a Platelet Function Analyzer PFA200 (Siemens Healthcare GmbH, Erlangen, Germany).

Urea Solubility test: 0.2 mL of plasma + 0.2 mL of 0.025 M CaCl_2_ were pipetted into a glass tube at 37ºC until clot formation. Then, 3 mL of 5 Mol/L Urea were added, the tube was capped and inverted, and the clot was inspected for its presence or absence at regular intervals.^13^

Acetic Acid Solubility test: 0.2 mL of plasma + 0.3 mL of saline + 0.1 mL of 30 IU/mL Thrombin were pipetted into a glass tube at 37ºC until clot formation. Then, 3 mL of 2% acetic acid were added, the tube was capped and inverted, and the clot was inspected for its presence or absence at regular intervals.^13^

To study clot lysis patient and normal whole blood as well as mixtures of patient’s and normal pooled plasma (NP), prepared to obtain increasing concentration of pFXIII, were used for TEG analysis (Thromboelastograph D Hellige, GMBH 1990, Germany). In each mixture pFXIII-A level, solubility tests and TEG with Ca^2+^ and with Ca^2+^ and 3.2 U/mL SK were also performed. For TEG Ca^2+^ 300 μL of whole blood or plasma were pipetted into the steel cuvette, and 50 μL of 0.11 M CaCl_2_ was added to start the TEG reaction. For TEG Ca^2+^ + SK, 290 μL of whole blood or plasma were pipetted into the steel cuvette, then 10 μL of an 112 U/mL SK solution was added, and at the end 50 μL of 0.11 M CaCl_2_ as starter reagent. It is important to note that SK at a subthreshold concentration (3.2 UI/ml), did not affect the TEG of normal plasmas.

Parameters analyzed were: reaction time R, the velocity of fibrin formation as α angule and time to a 20 mm of amplitude (k), Maximum amplitude (MA), elasticity (ɛ) and Lysis at 60 min (Lys 60). ɛ is calculated as 100 × MA/100-MA. TEG parameters were expressed as the mean of duplicates.

The study has been carried out in accordance with The Code of Ethics of the World Medical Association (Declaration of Helsinki) for experiments involving humans. Writing consent was signed by patient’s mother.

## Results

**Figure 1:**
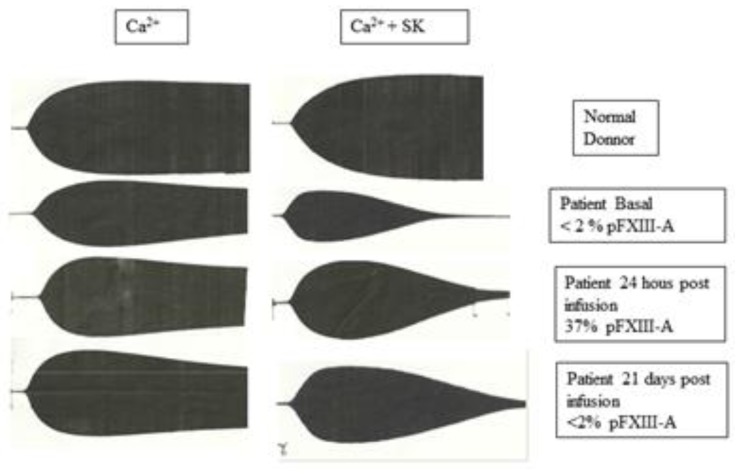
Whole blood TEGs of a normal control and patient’s samples at different time points: at diagnosis, 24 hours post the first replacement therapy and 21 days after the last replacement, 12 months from replacement therapy start.

Results of coagulation tests obtained from patient’s plasma and platelet function results are shown in Table 1. TEG of de patient’s whole blood at diagnosis was pathologic, with prolonged k, diminished α angle, MA and ɛ, and increased Lys 60. To test the lysis of the clot, the analysis of TEGs from normal, patient at diagnosis, at day one post-FXIII concentrate infusion and day 21 after one year of replacement therapy, were performed with or without SK(subthreshold concentration). Both basal and one-day post infusion TEG showed completely lysis with SK addition but not normal donor’s one (Figure 1). Parameters of TEGs from patient whole blood pre and day-1 post-FXIII infusion, with and without SK addition, as well as 21 days post infusion are shown in Table 2. As it can be seen, low pFXIII-A levels have not significant influence in R but affect k, MA, ɛ and Lys 60, except in 21 days post infusion sample after a one year period of prophylactic treatment, in which only Lys 60 was increased.

**Figure 2:**
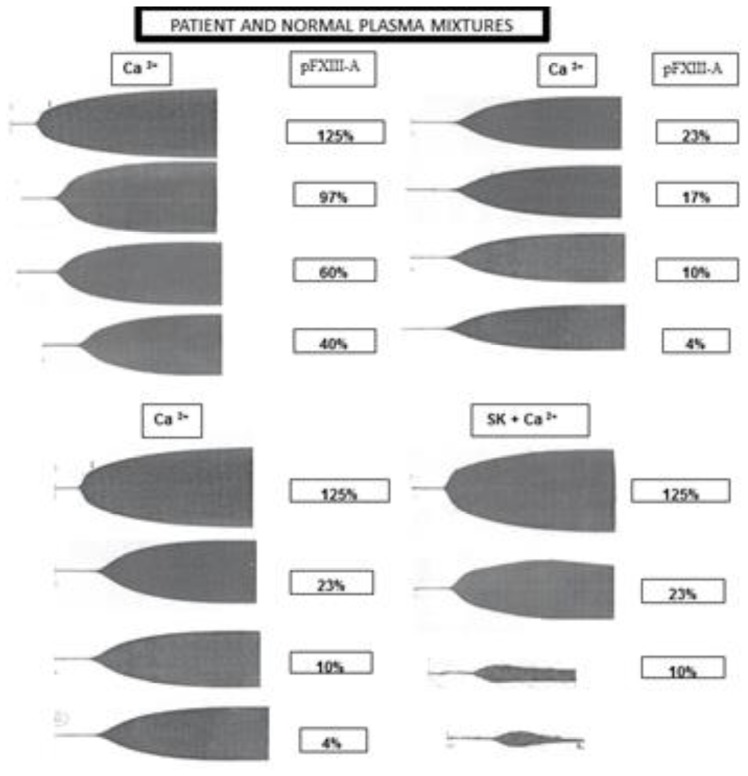
TEG graphics on mixtures of plasma from patient (at diagnosis) and normal donors, FXIII-A concentration between 4–125% at the upper panel. TEG Ca ^2+^ + SK graphics of 4 different mixtures are also shown in the lower panel.

TEG graphics of mixtures of plasma from patient (at diagnosis) and normal donors pooled plasma (4–125% of pFXIII-A) are shown in Figures 2. Results of solubility tests of these mixtures are shown in Table 3. The Ca-urea solubility was abnormal at pFXIII-A < 9%, thrombin-acetic acid at pFXIII-A <18%, but TEG graphics were abnormal at < 23% and Lys in TEG Ca^2+^ with SK at < 40% of pFXIII-A. In the sample of 21 days post infusion after one year of replacement therapy, solubility tests results were slightly altered, 7 hours for Thrombin-acetic acid, and 12 hours for Ca-urea, even when pFXIII-A levels measured were less than 2%.

**Figure 3:**
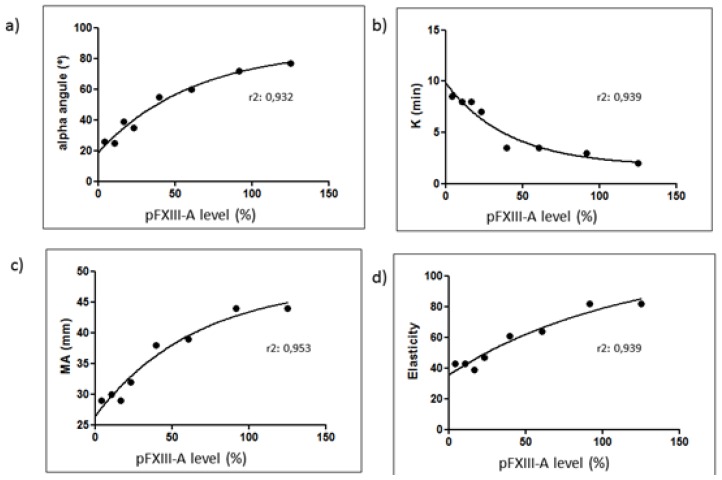
Relationship between TEG parameters α angle (a), k (b), MA (c) and ɛ (d), and FXIII-A concentration (4–125%) in plasma mixtures from patient (at diagnosis) and normal donors. Each point represents the mean of duplicate determinations.

There was a good correlation between α angle, k, MA and ɛ, with pFXIII-A levels in the mixtures (Figure 3 a, b, c and d), described by nonlinear regression equations.

When analyzing one congenital probably heterozygous (pFXIII-A level of 26%), and three acquired mild deficient (pFXIII-A 22–39%) patient’s whole blood samples we observed that MA and ɛ were decreased and Lys 60 was increased in TEG Ca^2+^ with SK (Table 4) compared with TEG Ca^2+^. Additionally, in two of these samples the values of MA and ɛ were slightly decreased, and one of them also had the Lys 60 in the high reference limit in TEG Ca^2+^. However, in all of them the solubility tests were negative (Time to lysis 5M Urea >16 hours, Time to lysis 2% Acetic Acid >12 hours).

## Discussion

Congenital FXIII deficiency, a rare autosomal recessive condition, and patients often suffer from umbilical stump bleeding, subcutaneous bleeding, muscle hemorrhage, postoperative hemorrhage, and potentially fatal intracranial hemorrhage.^1^,^4^,^12^ The case we described here suffered from umbilical stump bleeding, and subcutaneous and muscle bleedings at very early age.

FXIII deficiency is not detected by routine coagulation assays and needs to be searched by measuring pFXIII levels with quantitative assays. Traditional screening tests, the solubility of the fibrin clot in concentrated urea, acetic or mono chloroacetic acid solutions have been and are still used in developing countries like Iran and in about 20% of developed countries to screen for FXIII deficiency in addition to clinical manifestations and family history.^13^,^14^ As previously reported, these qualitative methods detect only homozygous or double heterozygous FXIII deficiency, are not standardized, and their sensitivity depends on the fibrinogen level, the clotting reagent (thrombin and/or Ca^2+^), and the type and concentration of the solubilizing agent. The detection limit of the clot solubility assay varies between less than 1% and 10–18% pFXIII activity, depending on the assay characteristics.^10^,^15^,^16^ The lack of sensitivity of these tests may contribute to undiagnosed or late-diagnosed FXIII deficiencies^17^ as has been demonstrated in some provinces of southeast Iran. In this region, the prevalence of the FXIII deficiency greatly increased when the molecular analysis of the gene sequence, particularly at exon 4, where is found the most common mutation (Trp187Arg), was implemented.^13^–^14^ This diagnosis is important because, among the rare bleeding disorders, homozygous FXIII deficiency is associated with the highest proportion of severe bleeding episodes, like the potentially fatal intracranial hemorrhage occurring in approximately one-third (34 %) of patients with severe deficiency.^4^ Due to this lack of sensitivity and standardization,^10^,^15^ the solubility tests are currently not recommended.^6^ The use of a quantitative functional pFXIII activity assay is considered as the first line screening test because detects all forms of FXIII deficiency,^6^ but the most widely used, the ammonia-release assay has a low sensitivity with a quantitation limit of 5%. 17 In our case, FXIII deficiency was diagnosed by immunological measurement of pFXIII-A subunit and solubility tests. However, mild congenital and acquired additional deficient patients were not detected by solubility tests.

The influence of FXIII on clot strength measured by TEG or thromboelastometry has been described in some previous studies in congenital and acquired FXIII deficiency.^18^^–21^ Moreover, it has been used in the follow-up of patients with homozygous or double heterozygous deficiency on prophylactic therapy with recombinant FXIII-A_2_.^22^ In concordance, the TEG Ca ^2+^ on our patient’s whole blood was abnormal and improved after plasma-derived FXIII was administered.

We assessed the effect of FXIII on TEG induced by calcium in mixtures of normal pool plasma with patient’s FXIII deficient plasma and confirmed that most affected parameters were MA and ɛ, as well as Lys 60 when pFXIII-A levels were below 23%. These results agreed to those shown by Nielsen and col.^20^

Additionally, we also tested the clot lysis by challenged the TEG with subthreshold concentrations of SK and demonstrated that it was exacerbated when the pFXIII concentration was below 40%. This test could be potentially useful as a screening test for heterozygous or mild acquired FXIII deficiency, instead of solubility tests. Our findings would also explain some bleeding manifestations observed in patients with FXIII congenital heterozygous deficiency, like bleeding post-surgery, post tooth extraction, menorrhagia, postpartum hemorrhage^7^–^10^ or acquired deficiencies.^10^–^13^^,21,23,24,25^ In fact, in some of them, a lower clot strength and an increased lysis were demonstrated.^20^^,21,24^ A rationale for FXIII replacement in post cardiovascular surgery setting has been reported..^21^ In our study, we observed TEG MA and ɛ alterations and exacerbated lysis in TEG Ca ^2+^ + SK of samples from one congenital probably heterozygous, and 3 acquired post-surgery FXIII deficient patients. The TEG parameters alterations are in agreement to those observed in post-surgery patients in different settings^21,23–24^ and in “in vitro” studies.^26,27^

TEG graphic and parameters (MA and ɛ) and the clot disappearance time in solubility tests were slightly affected in the 21 days post infusion sample after one-year prophylactic therapy in contrast to that seen in sample at diagnosis, even when the measured pFXIII-A were <2% in both samples. One possible explanation could be that FXIII-A levels are different between samples being baseline one (at diagnosis) almost 0 % and 2% or even more in the 21 days sample, and the limit of quantification of the test do not allow distinguishing between them. It has been reported a higher variability and inaccuracy in samples after FXIII replacement in a NEQAS study.^15^ The improvement in functional tests (Solubility and TEG) observed in the 21 days post infusion sample is in agreement to the clinical evolution of the girl, who has not experienced bleeding symptoms under prophylactic therapy.

In conclusion, TEG parameters, particularly MA, ɛ and Lys60 induced by SK seemed to be more sensitive to low levels of FXIII than solubility tests, being probably a better tool for FXIII deficiency diagnosis and replacement therapy follow up in laboratories were quantitative pFXIII assays are not available. Although this work was based on experimental data obtained from a single severe case, and few mild deficient cases, the high sensitivity of clot lysis at a subthreshold concentration of SK could be considered as one of the possible explanation for the bleeding manifestations frequently reported after hemostatic challenges in many patients with pFXIII levels between 5–40%.

## Figures and Tables

**Table 1:** Patient’s results at diagnosis.

Test	Result	RV
PT (% ACT)	91	70–120
APTT (sec)	33	24–40
TT (sec)	13	13–16
Fibrinogen (mg/dL)	263	180–400
VIIIc (UI/dL)	101	50–150
vWF:Ag (%)	99	50–150
vWF:Ab (%)	97	50–150
5M Urea solubility (min)	50	>24 horas
2% Acetic Acid Solubility (min)	45	>12 horas
pFXIII-A (%)	< 2	60–160
PFA COL/EPI (seg)	92	70–148
PFA COL/ADP (seg)	83	60–102
LTA Aggregation amplitud (%)		
*Collagen 1μg/ml*	86	75–100
*ADP 3μM*	73	69–100
*Epinephrin 50μM*	72	68–100
*Arachidonic Acid 0.7 mM*	76	70–100

Abbreviations: PT prothrombin time, APTT activated platelet thromboplastin time, TT thrombin time, VIIIc factor VIIIc level, vWF: Ag von Willebrand factor antigen, vWF:Ab von Willebrand factor activity, pFXIII-A plasma factor XIII A subunit, PFA platelet function analyzer, LTA Light transmission aggregometry, ADP Adenosine diphosphate.

**Table 2:** Parameters of TEGs from patient whole blood at diagnosis, 1 day post FXIII infusion, as well as 21 days post infusion, with and without SK addition.

	FXIII-A	R		α		k		MA		ɛ		Lys 60	
		Ca^2+^	Ca^2+^ + SK	Ca^2+^	Ca^2+^ + SK	Ca^2+^	Ca^2+^ + SK	Ca^2+^	Ca^2+^ + SK	Ca^2+^	Ca^2+^ + SK	Ca^2+^	Ca^2+^ + SK
**N**	125%	5	5	63	63	8	8	60	52	150	108	0	4
**P** at diagnosis	<2%	8	8	50	53	14	13	39	***24***	64	***32***	23	***100***
**P** 1 day post infusion	37%	8	7	65	65	11	10	52	***46***	108	***85***	31	***76***
**P** 21 days post infusion	<2%	6	7	66	56	10	12	60	***41***	150	***69***	25	***69***

All values represent the mean of duplicate determinations. Abbreviations: R: reaction time; α: velocity of thrombus formation; k: time to a 20 mm of amplitude; MA: Maximum amplitude; ɛ: elasticity and Lys 60: Lysis at 60 min. Ca^2+:^ TEG started with CaCl_2_ 0.018 M; Ca^2+^ + SK: TEG started with CaCl_2_ 0.018 M and 3.2 UI/ml Streptokinase.

**Table 3:** Solubility test results and TEG parameters of plasma from patient (at diagnosis) and pooled normal plasma mixtures, range of pFXIII-A concentration between 4–125%.

pFXIII-A level (%)	Ca2+/ Urea (h)	Thrombin/Acetic acid (h)	TEG Ca^2+^				TEG Ca^2+^ + SK
			κ min	α º	MA mm	ɛ	LYS60
<2	0,75	0,1	17	25	29	41	100
9,4	>16	0,75	17	26	29	41	61
18,5	>16	>12	15	36	32	47	-
23	>16	>12	9	39	36	56	31
37	>16	>12	9	62	38	61	21
45	>16	>12	8	69	39	64	-
125	>16	>12	4	80	44	79	0

All values represent the mean of duplicate determinations. Abbreviations: α angule: velocity of thrombus formation; k: time to a 20 mm of amplitude; MA: Maximum amplitude; ɛ: elasticity.

**Table 4:** Parameters of TEGs of whole blood samples from additional patients with heterozygous or mild acquired deficiencies.

	pFXIII-A	R		α		k		MA		ɛ		Lys 60	
		Ca^2+^	Ca^2+^ + SK	Ca^2+^	Ca^2+^ + SK	Ca^2+^	Ca^2+^ + SK	Ca^2+^	Ca^2+^ + SK	Ca^2+^	Ca^2+^ + SK	Ca^2+^	Ca^2+^ + SK
**N**	125%	5	5	63	63	8	8	60	52	150	108	0	4
**Heterozygous**	26%	7	7	64	61	10	11	60	***50***	150	***100***	02	***27***
**Acquired deficient 1**	22%	8	8	50	47	10	12	50	***46***	100	***85***	15	***45***
**Acquired deficient 2**	33%	8	7	65	65	9	11	50	***43***	100	***75***	10	***28***
**Acqired deficient 3**	39%	6	7	66	58	9	10	60	***49***	150	***92***	9	***18***

All values represent the mean of duplicate determinations. Abbreviations: R: reaction time; α: velocity of thrombus formation; k: time to a 20 mm of amplitude; MA: Maximum amplitude; ɛ: elasticity and Lys 60: Lysis at 60 min. Ca^2+:^ TEG started with CaCl_2_ 0.018 M; Ca^2+^ + SK: TEG started with CaCl_2_ 0.018 M and 3.2 UI/ml Streptokinase.
